# The Interleukin-20 Cytokine Family in Liver Disease

**DOI:** 10.3389/fimmu.2018.01155

**Published:** 2018-05-28

**Authors:** Esther Caparrós, Rubén Francés

**Affiliations:** ^1^Departamento de Medicina Clínica, Universidad Miguel Hernández, San Juan de Alicante, Spain; ^2^Instituto ISABIAL-FISABIO, Hospital General Universitario de Alicante, Alicante, Spain; ^3^CIBERehd, Instituto de Salud Carlos III, Madrid, Spain

**Keywords:** interleukin-20 family, viral hepatitis, alcoholic liver disease, non-alcoholic liver disease, hepatocellular carcinoma

## Abstract

The three main causes of inflammation and chronic injury in the liver are viral hepatitis, alcohol consumption, and non-alcoholic steatohepatitis, all of which can lead to liver fibrosis, cirrhosis, and hepatocellular carcinoma, which in turn may prompt the need for liver transplant. The interleukin (IL)-20 is a subfamily part of the IL-10 family of cytokines that helps the liver respond to damage and disease, they participate in the control of tissue homeostasis, and in the immunological responses developed in this organ. The best-studied member of the family in inflammatory balance of the liver is the IL-22 cytokine, which on the one hand may have a protective role in fibrosis progression but on the other may induce liver tissue susceptibility in hepatocellular carcinoma development. Other members of the family might also carry out this dual function, as some of them share IL receptor subunits and signal through common intracellular pathways. Investigators are starting to consider the potential for targeting IL-20 subfamily members in liver disease. The recently explored role of miRNA in the transcriptional regulation of IL-22 and IL-24 opens the door to promising new approaches for controlling the local immune response and limiting organ injury. The IL-20RA cytokine receptor has also been classified as being under miRNA control in non-alcoholic steatohepatitis. Moreover, researchers have proposed combining anti-inflammatory drugs with IL-22 as a hepatoprotective IL for alcoholic liver disease (ALD) treatment, and clinical trials of ILs for managing severe alcoholic-derived liver degeneration are ongoing. In this review, we focus on exploring the role of the IL-20 subfamily of cytokines in viral hepatitis, ALD, non-alcoholic steatohepatitis, and hepatocellular carcinoma, as well as delineating the main strategies explored so far in terms of therapeutic possibilities of the IL-20 subfamily of cytokines in liver disease.

## The Interleukin (IL)-20 Cytokine Family: Molecular Features and Role in Disease

The IL-20 subfamily of cytokines represents one of the three subfamily groups comprising the IL-10 family of cytokines. This family also includes the IL-10 cytokine itself and the type III IFN group (with IL-28A, IL-28B, and IL-29 members), categorized according to their biological function (1). They all work together to maintain epithelial tissue homeostasis and integrity, enhancing innate epithelial immunity, and regulating the healing process after infection or inflammatory events (2–4). The IL-20 subfamily includes IL-19, IL-20, IL-22, IL-24, and IL-26, which all have the common function of communicating leukocytes and epithelial cells in different tissues such as the liver. They play an important role in controlling tissue regeneration following injury, promoting survival as well as inhibiting apoptosis of epithelial cells (5).

**Figure 1:**
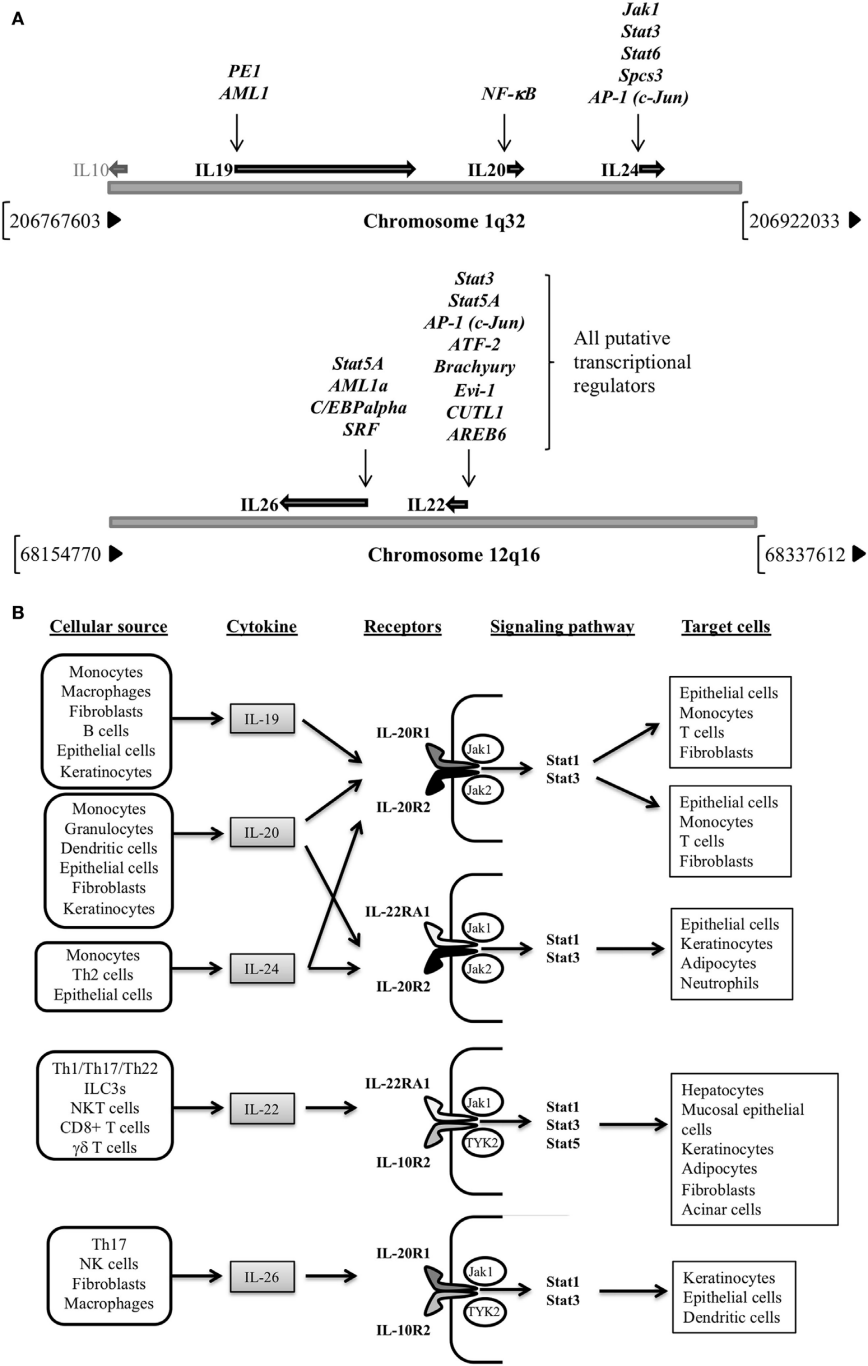
(A) Genomic location for IL-20 subfamily of cytokines genes (chromosomes 1q32 and 12q16) and their transcriptional regulators. Note that for IL-22 and IL-26 putative regulators are provided (Human Gene Database, Weizmann Institute of Science, https://genecards.weizmann.ac.il/v3). (B) Cellular sources, receptors, signaling pathways activated and target cells of the IL-20 subfamily of cytokines.

The IL-20 subfamily of cytokines is encoded by genes located in different clusters, which all share genomic organization, primary and secondary structures, and receptor complexes (6). IL-19, -20, and -24 genes are confined in chromosome 1q32, close to IL-10 gene location (7), while IL-22 and IL-26 are enclosed in chromosome 12q16 (8, 9) (Figure 1A). Regarding their regulation, different transcription factors have been reported for IL-19 (PE1 and AML-1) (10), IL-20 (NF-κB) (11), and IL-24 (Jak1, Stat3, Stat6, Spcs3, and AP-1) (12, 13), though only putative regulators have been suggested for IL-22 and IL-26 transcriptional control. Signaling events by IL-20 subfamily members result in receptor dimerization; Janus kinase (Jak) 1, Jak2, and Tyrosine kinase 2 phosphorylation; and final signal transducer and activator of transcription (STAT)1 STAT3, and STAT5 activation (14–17) (Figure 1B). The best-studied cytokine signaling pathways are those regulated by IL-10 and IL-22, with the mitogen-activated protein kinases’ final recruitment for anti-apoptotic and mitogenic gene expression in target cells.

Cellular sources of the IL-20 subfamily of cytokines include monocytes, macrophages (18–20), dendritic cells (DCs) (21–23), B-cells (22), T helper (Th) 2 and Th17 cells, cytotoxic CD8+ T cells, natural-killer (NK) cells, innate lymphoid cell (ILC) 3 (4, 24–36), fibroblasts (37), NKT cells (38–40), and γδ T cells (41–43) to epithelial cells (12, 44–48) (Figure 1B). They regulate new cytokine secretion according to their cellular targets which are immune system cells, such as monocytes, DCs, neutrophils or T cells (16), and also hepatocytes (2, 49), acinar cells (50), fibroblasts, epithelial cells (51–53), keratinocytes (54), or adipocytes (4, 55).

In the liver, the IL-20 cytokine subfamily has a key role in inflammatory pathological processes. The best-studied member of the family in liver homeostasis is the IL-22 cytokine. In a mouse model, it has been shown to lessen metabolic syndrome—a condition related to chronic low-level inflammation—by inducing the activation and expression of lipogenesis-related genes and helping with the triglyceride and cholesterol metabolism (56). IL-22 also prevents apoptosis of hepatic stellate cells and attenuates liver fibrosis in mice (57, 58) and rat models (59), and reported as a predictive severity marker in advanced stages of liver cirrhosis (60). Due to its hepatoprotective and anti-fibrotic properties, different studies have proposed IL-22 as a plausible candidate in the treatment of alcoholic liver disease (ALD) (61, 62). Commensal bacteria, such as *Lactobacillus*, have been shown to induce the production of IL-22 by gut ILCs. Rising IL-22 levels provoke the recruitment of regulatory DCs into the liver, constricting the hepatic inflammatory response, and favoring a tolerant tissue microenvironment (63). It is still unknown whether the protective role attributed to IL-22 is unique or shared with other IL-20 family members, but IL-19, IL-20, and IL-24 may also function as protective cytokines during liver inflammation (3), as they all share signaling pathways through the IL-20R2 receptor subunit, which is greatly induced during LPS liver challenge (64). IL-20 and IL-24 can also signal through heterodimeric receptor formation with IL-22RA1, which could entail some redundancy in their action. Both cytokines participate in lipid metabolism regulation, although they have failed to improve metabolic disorder in obese mice (65). Also, IL-19, IL-22, and IL-24 participate in wound healing (66–69), an event present not only in the skin but also in liver disease as a first step for fibrogenesis, cirrhosis, and liver failure (70). IL-22 also induces the expression of vascular entothelial growth factor A, which facilitates angiogenesis and neo-vacularization during wound healing (66). IL-24 improves resolution of *Salmonella typhimurium* infection when exogenously administered to mice (71), a quality not confirmed up to now for the IL-20 cytokine. Furthermore, IL-24 has been shown to inhibit hepatoma cell growth in the mouse model (10). Finally, IL-26 has antimicrobial and antiviral functions (72, 73). Its role in liver disease is mainly limited to hepatitis C virus (HCV) infection, although it is also implicated in liver fibrosis, as shown in a mouse model of obliterative bronchiolitis (74). IL-26 is overexpressed in HCV infection, inducing an antiviral state and endowing NK cells with a higher killing capacity over HCV-infected cells (75). IL-26 also targets epithelial cells, probably favoring a better cutaneous and mucosal immunity (76). Table 1 summarizes the functions of the IL-20 subfamily of cytokines in liver disease.

**Table 1:** Roles of interleukin (IL)-20 subfamily cytokine members in liver diseases.

Liver condition	IL-20 cytokine family member involved	Role	Reference
HBV	IL-22	Downregulation of viral proteins	(77)
		Promotion of cell proliferation, neutrophil recruitment, induction of chemokine production	(78)
		Induction of liver progenitor cells	(79)
		Chronic liver inflammation and fibrosis	(80)
		Proinflammatory	(81)

HCV	IL-22	Pathological	(82, 83)
		Hepatoprotective	(84, 85)

	IL-26	Induction of antiviral state and improvement of killing activity by NK cells	(76)

ALD	IL-22	Correlation with liver damage progression	(86)
		Good prognostic marker	(87)
		Hepatoprotective	(61, 62, 88, 89)

NAFLD/NASH	IL-20	Progression of hepatic insulin resistance, regulation of lipid metabolism	(56, 65, 90)
	IL-22	Regulation of lipid metabolism and inflammation	(56, 65, 91, 92)
	IL-24	Regulation of lipid metabolism	(56, 65)
HCC	IL-22	Promoter of tumor growth	(93, 94)
		Negative prognostic marker	(95)

	IL-24	Inhibition of cell growth	(96)
	IL-26	Upregulation of NK anti-tumor activity	(75)

Liver inflammation	IL-19, IL-20, IL-22, IL-24	Protective during liver inflammation	(3, 43, 57)

Liver fibrosis	IL-22	Amelioration of liver fibrosis	(58, 59, 97)
		Predictor of severity (cirrhosis)	(60)

*Salmonella typhimurium* infection	IL-24	Resolution improvement	(71)

*HBV, hepatitis B virus; HCV, hepatitis C virus; ALD, alcoholic liver disease; NAFLD/NASH, non-alcoholic fatty liver disease/non-alcoholic steatohepatitis; HCC, hepatocellular carcinoma*.

## IL-20 Cytokine Family in Viral Hepatitis

Hepatitis B virus (HBV) and HCV are the most frequent virus types that induce chronic or acute and chronic forms of hepatitis, respectively. In patients, there is an increase in the number of cells producing IL-22 in both HBV and HCV (98–100). Furthermore, there is an increase in the number of liver progenitor cells in infected mice, a finding that has been corroborated in patients with chronic HBV (79). IL-22 production in HBV-infected patients seems to be under Notch pathway control. Experiments performed in a mouse model *in vivo* with Notch signaling inhibitors show that IL-22 was clearly diminished in the liver (101). It has also been reported to promote the modulation of the immune response during viral infection by downregulating the S100 family of viral proteins (77). Nevertheless, a dual role in protection and inflammation induction has been proposed for IL-22 in chronic HBV infection, either promoting cell proliferation and improving fibrosis, or inducing chemokine production and neutrophil recruitment (78). More recently, different controversial roles have emerged for IL-22 in viral infection. For example, IL-22 has been associated with liver fibrosis severity in patients infected with HCV (82, 83), with a possible pathological role residing in the accumulation of IL-22 in fibrotic areas due to its role in ameliorating liver tissue damage (85). In this sense, Sertorio et al. found that IL-22 functions as a protective factor, while IL-22 binding protein, a natural protein antagonist for IL-22, contributes to worsening liver fibrosis in chronic HCV infection (84). On the other hand, Zhao et al. attributes a pathogenic role to IL-22 because of its participation in promoting Th17 recruitment in chronic liver inflammation and fibrosis in HBV infection (80), Zhang et al. postulates that IL-22 works as a proinflammatory cytokine in response to HBV (81), and Gao et al. raise the possibility that the inflammatory tissue milieu represents different scenes for different functions of IL-22 (102). More studies are needed to shed light on the IL-22 function in liver fibrosis, but this cytokine may be considered a therapeutic opportunity for future clinical management of liver disease.

Another IL-20 family member implicated in HCV infection is IL-26. This cytokine is overexpressed in serum when HCV infection occurs, and it is detected in liver lesions in chronic infection, particularly in patients with severe liver inflammation. As previously indicated, IL-26 boosts NK cell response to HCV challenge through the upregulation of tumor necrosis factor (TNF)-related apoptosis-inducing ligand (TRAIL) expression in the membrane surface of NK cells (75). Therefore, IL-26 has been proposed as a marker of inflammation in chronic HCV infection.

## IL-20 Cytokine Family in ALD

Alcoholic liver disease comprises different categories of liver alterations, from liver steatosis to cirrhosis or cancer. In this context, alcohol consumption induces hepatotoxicity through intermediaries of ethanol metabolism, or by generating different mediators of the inflammatory response, like products of the oxidative stress response or proinflammatory cytokines secreted by liver Kupffer cells, such as TNF-α (103). Serum concentrations of both IL-17 cytokine protein isoforms, together with IL-22 levels, have been proposed as correlates of liver damage progression in ALD (86). Moreover, high-IL-22 levels seem to be a good prognostic marker (87). Liu et al. also describe an increase of IL-22 cytokine levels in alcoholic hepatitis patients, produced by macrophage *in vitro*, suggesting that the cytokine may have a hepatoprotective function (89). The authors also show an improvement in hepatocyte function and survival from ethanol-induced cell death when exposed to IL-22. Moreover, in a murine binge-drinking model, alcoholic liver injury is improved after treatment with IL-22 recombinant protein (88).

As both inflammation and hepatocyte damage are the main reasons for ALD, combined anti-inflammatory plus hepatoprotective therapy could hold promise for clinical management. At the time of writing, two different clinical trials on alcoholic hepatitis disease were in the recruitment phase (NCT01918462 and NCT02655510). Both trials implicate IL-22 in alcoholic hepatitis, with the first studying its biological effects in the disease and the second aiming to use recombinant human IL-22 to take advantage of the anti-steatotic and anti-apoptotic functions of this cytokine. These two pioneering clinical approaches may usher in new opportunities for handling ALD.

## IL-20 Cytokine Family in Non-ALD

Non-alcoholic fatty liver disease (NAFLD) comprises a range of pathologies, from steatosis to non-alcoholic steatohepatitis (NASH). The mechanisms underlying this aggravation remain undefined, but a title role has been given to the innate immunity, inflammatory cytokines (85), and commensal microbiota (104). The protective role of the intestinal mucus coat has been analyzed in a murine model of fatty liver disease [high-fat diet (HFD)]. The absence of mucin-2 (muc2) in muc2-deficient HFD-fed mice causes the activation of the mucosal immune system resulting in higher plasmatic and intestinal levels of IL-22, with a role regulating lipid metabolism and inflammation in the liver and adipose tissue (65, 91, 92).

In this line, investigators have detected high levels of Th22 cells in the liver tissue of IL-17-deficient mice, inferring a protective role during NASH progression (105). Regarding these Th cell populations, Rolla et al. point to the Th17/Th22 offset as a very important consideration when assessing NASH.

Increased IL-20 levels are present in visceral adipose tissue of NASH-diagnosed patients, and they are considered a target for miR-26a. This would induce a reduction in the expression of IL-20 cytokine (90). Estep et al. have studied IL-20 receptors as well as the cytokine itself, observing that IL-20RA is located in pericellular areas of fibrotic zones and that the expression of IL-20RB is also upregulated in NASH patients. This proinflammatory cytokine signals through STAT3 transcription factor, also activating the downstream IL-22 or IL-10 signaling pathway. STAT3 has been directly implicated in the progression of hepatic insulin resistance and its polymorphisms linked to NAFLD advancement (106, 107), so it could be a potential objective for modulating cytokine response during NAFLD development.

## IL-20 Cytokine Family in the Progression of Hepatocellular Carcinoma

Hepatocellular carcinoma (HCC) is the most frequent liver cancer variant, with incidence increasing every year. IL-22 is a well-established hepatoprotective cytokine; it promotes liver healing and tissue repair, preventing cellular apoptosis (62). It has a dual role in the control of liver disease, with new evidence showing that it participates in controlling viral and alcohol-induced HCC (108). Nonetheless, IL-22 can also promote tumor growth both *in vitro* and *ex vivo* (93, 94), and its levels are increased in patients with hepatocellular carcinoma (95). In a mouse model of HCC, metformin has been shown to reduce tumor growth and to inhibit the IL-22 signaling pathway (109). These data suggest that IL-22 might enhance liver tissue susceptibility to HCC development. Thus, although these results have been obtained from the animal model, clinicians must consider the risk of developing liver cancer when making therapeutic decisions for patients with hepatic disease in the future.

Interleukin-24 has been shown to inhibit HCC cells metastasis (96). This cytokine has been recently described to be regulated by the onco-microRNA miT-203a-3p.1. This microRNA controls IL-24 expression, and its inhibition can reverse HCC cell proliferation and metastasis (110). In another cellular context, IL-24 acts synergistically with Notch pathway inhibitors in reducing tumor cell invasion and migration of HepG2 liver cancer cell line (111). In combination, the Notch pathway, miRNA miT-203a-3p.1, and IL-24 can be now considered possible therapeutic objectives in HCC management.

An *in vitro* anti-tumor effect has also been described for IL-26 due to the fact that NK cell-specific cytotoxicity against the hepatocellular carcinoma line HuH7.5 is highly upregulated in the presence of IL-26 (75). The induction mechanisms for this upregulation depend on the overexpression of the TRAIL receptor in NK cells, which contributes to their cytotoxic activity.

## Future Directions and Concluding Remarks

The different cytokine members of the IL-20 subfamily perform an array of antiviral, anti-apoptotic, progenitor cell-generating, and lipid metabolism regulator functions as a part of liver homeostasis in viral hepatitis, ALD, NASH, and hepatocarcinoma. The protective role of this family preventing tissue damage and inflammation makes these cytokines crucial targets for liver disease therapy: (a) IL-22 has shown both pathological and hepatoprotective roles, so IL-22-IL-22R1 therapeutic possibilities seem to depend on the presence of different inflammatory conditions, which may either require the activation of this pathway or justify its inhibition; (b) IL-19, IL-20, and IL-24 have a key role in the wound healing process of fibrogenesis in the liver, and IL-22 in the angiogenesis and neo-vascularization events; (c) IL-26 can be considered a promising scope in infectious diseases management, given the antimicrobial and antiviral function already reported for this cytokine.

Our review hints at a wide and still unexplored area of research on the close interactions between ILs and the pathophysiology of liver diseases. Taking account of the number of processes in which IL-20 subfamily of cytokines is actively participating, therapeutic interventions with these cytokines stand out as a vast and promising area of study.

## Author Contributions

EC and RF equally contributed to the design and writing of the manuscript.

## Conflict of Interest Statement

The authors declare that the research was conducted in the absence of any commercial or financial relationships that could be construed as a potential conflict of interest.
